# Phospholipase C-related but catalytically inactive protein modulates pain behavior in a neuropathic pain model in mice

**DOI:** 10.1186/1744-8069-9-23

**Published:** 2013-05-02

**Authors:** Tomoya Kitayama, Katsuya Morita, Rizia Sultana, Nami Kikushige, Keisuke Mgita, Shinya Ueno, Masato Hirata, Takashi Kanematsu

**Affiliations:** 1 Department of Cellular and Molecular Pharmacology, Division of Basic Life Sciences, Institute of Biomedical and Health Sciences, Hiroshima University, 1-2-3 Kasumi, Minami-ku, Hiroshima 734-8553, Japan; 2 Department of Neurophysiology, Hirosaki University Graduate School of Medicine, 5 Zaifucho, Hirosaki, Aomori 036-8562, Japan; 3 Laboratory of Molecular and Cellular Biochemistry, Faculty of Dental Science, Kyushu University, Fukuoka 812-8582, Japan

**Keywords:** KCC2, GABA_A_ Receptor, Neuropathic pain, Partial sciatic nerve ligation, PRIP

## Abstract

**Background:**

An inositol 1,4,5-trisphosphate binding protein, comprising 2 isoforms termed PRIP-1 and PRIP-2, was identified as a novel modulator for GABA_A_ receptor trafficking. It has been reported that naive *PRIP-1* knockout mice have hyperalgesic responses.

**Findings:**

To determine the involvement of PRIP in pain sensation, a hind paw withdrawal test was performed before and after partial sciatic nerve ligation (PSNL) in *PRIP-1* and *PRIP-2* double knockout (DKO) mice. We found that naive DKO mice exhibited normal pain sensitivity. However, DKO mice that underwent PSNL surgery showed increased ipsilateral paw withdrawal threshold. To further investigate the inverse phenotype in *PRIP-1* KO and DKO mice, we produced mice with specific siRNA-mediated knockdown of *PRIP*s in the spinal cord. Consistent with the phenotypes of KO mice, *PRIP-1* knockdown mice showed allodynia, while *PRIP* double knockdown (DKD) mice with PSNL showed decreased pain-related behavior. This indicates that reduced expression of both PRIPs in the spinal cord induces resistance towards a painful sensation. GABA_A_ receptor subunit expression pattern was similar between *PRIP-1* KO and DKO spinal cord, while expression of K^+^-Cl^-^-cotransporter-2 (KCC2), which controls the balance of neuronal excitation and inhibition, was significantly upregulated in DKO mice. Furthermore, in the DKD PSNL model, an inhibitor-induced KCC2 inhibition exhibited an altered phenotype from painless to painful sensations.

**Conclusions:**

Suppressed expression of PRIPs induces an elevated expression of KCC2 in the spinal cord, resulting in inhibition of nociception and amelioration of neuropathic pain in DKO mice.

## Findings

### Background

We identified a d-*myo*-inositol 1,4,5-triphosphate-binding protein with a domain organization similar to phospholipase C-δ, but with no enzymatic activity. We therefore termed this protein phospholipase C-related but catalytically inactive protein (PRIP) [1-3]. PRIP exists in 2 subtypes and can bind to GABA_A_ receptor β subunits [4], GABA_A_ receptor associated protein [5], and protein phosphatase 1 and 2A [6,7].

Naive *PRIP-1* knockout (KO) mice demonstrate a marked decrease in the withdrawal threshold in the von Frey hair test because of altered expression of GABA_A_ receptor subunit in their central nervous system [8]. In the present study, we investigated the role of PRIP-1 and PRIP-2 in pain sensation using *PRIP-1* and *PRIP-2* double knockout (DKO) mice, and *PRIP-1* and/or *PRIP-2* knockdown (KD) mice.

### Materials and methods

#### Animals

Ten- to fourteen-week-old male *PRIP-1* KO [5,8] and DKO [7,9] mice, in a C57BL/6J mouse background, and ddY mice were used. All procedures and handling of animals were performed with permission according to the guidelines of Hiroshima University.

#### Seltzer model and paw withdrawal threshold test

Partial sciatic nerve ligation (PSNL) was performed according to the procedure described by Seltzer et al. [10]. A paw withdrawal threshold in response to probing with von Frey hair (gram weight to buckling) was measured.

#### Generation of PRIP knockdown mice by intrathecal injection with siRNA

Three siRNA target sequences for each *PRIP-1* and *PRIP-2* gene were designed using a manufacturer-provided software (see Table 1). Synthetic siRNAs (0.45 pmol [0.15 pmol for each]/5 μl/animal, purchased from iGENE, Therapeutics Inc., Tokyo, Japan) were injected into the subarachnoid space between L5 and L6 vertebrae of mice using hemagglutinating virus of Japan envelope (HVJ-E) vector system (GenomeONE; Ishihara Sangyo Kaisha, Ltd., Osaka, Japan) [11].

**Table 1:** The sequences used in siRNA knockdown methods

**Target gene**	**Sequences of the siRNA oligonucleotide (sense)**
*PRIP1*	#1: 5′-GGAAGAAAGUUCGAGAAUACACCAU-AG-3′
	#2: 5′-GCGAGAAACUUUAUACAGAAGCACC-AG-3′
	#3: 5′-GAUAGAGGGUUCACUGGUUUCACAG-AG-3′
*PRIP2*	#1: 5′-GGACAAAGCUGGUACUGAAAUCACA-AG-3′
	#2: 5′-GCAGGAGCGUUGAAUUAGAUGUGUG-AG-3′
	#3: 5′-GCCGGAGCAGCAUCAUCAAGGAUGG-AG-3′

#### Immunoblot analysis

The region of L5 and L6 vertebrae, into which siRNAs were injected, was homogenized at 3 days postinjection with a homogenization buffer, and whole-cell fractions (for PRIPs, K^+^-Cl^-^-cotransporter-2 [KCC2], Na^+^-K^+^-Cl^-^-cotransporter-1 [NKCC1], glycine receptor [GlyR] α1, and tubulin) or cell membrane fractions (for GABA_A_ receptor subunits) were obtained [12]. The homogenates were subjected to SDS-PAGE followed by immunoblotting using specific primary antibodies of interest. Antibodies used are as follows: anti-PRIP-1 antibody [5], anti-PRIP-2 antibody [13], anti-β tubulin (Thermo Scientific, CA), anti-GABA_A_ receptor α1 subunit, anti-NKCC1 (Alpha Diagnostic International, TX), anti-GABA_A_ receptor α2 subunit (Aviva Systems Biology, CA), anti-GABA_A_ receptor α5 subunit (R&B Systems, MN), anti-GABA_A_ receptor α6 subunit (Imgenex, CA), anti-GABA_A_ receptor α4, β2/3, and γ2 subunit, anti-GlyRα1 (Merck Millipore, MA), anti-KCC2 (Santa Cruz Biotechnology, CA), and anti-phosphoserine (Acris antibodies, CA) antibodies. An enhanced chemiluminescence western detection system (Nacalai Tesque Inc., Kyoto, Japan) was used for development (ImageQuant™ LAS 4000 mini detection system; GE Healthcare Japan).

#### Statistical analyses

The density of each band was analyzed using NIH ImageJ software, and the densitometric units were corrected for tubulin. The data were expressed as the mean ± S.E.M. Statistical analyses are described in the figure legends.

## Results and discussion

**Figure 1:**
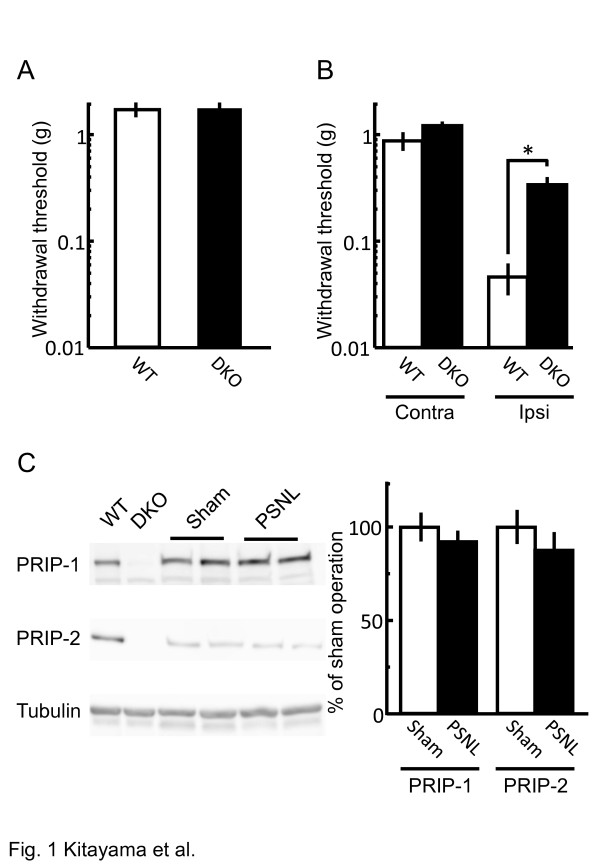
**Pain-related behavior in WT and DKO mice.** (**A**, **B**) Paw withdrawal threshold in the von Frey hair test was measured 10 days after nerve injury by using naive mice (**A**) or neuropathic pain model mice (**B**). The thresholds of PSNL-operated contralateral (Contra) and ipsilateral (Ipsi) sides were assessed in (**B**). Column chart shows withdrawal threshold in WT (open column) and DKO (closed column) mice (mean ± S.E.M., *n* = 7–10). *P < 0.05 versus the corresponding WT values (Student’s *t*-test). (**C**) Alteration of spinal PRIP-1 and PRIP-2 expression 10 days after sham operation (open column) or PSNL (close column) in naive C57BL/6 mice assessed by immunoblotting. The levels of immunoreactivity were normalized to that of β-tubulin and represented as % induction compared with the values of WT mice (means ± S.E.M., *n =* 4).

To examine pain-related behavior in DKO mice, PSNL was performed, and the withdrawal threshold of the hind paw was measured by applying von Frey filaments. Naive DKO mice had normal sensation levels in terms of withdrawal threshold (Figure 1A). This differed greatly from the significant reduction in the withdrawal threshold observed in *PRIP-1* KO mice [8]. After PSNL, the withdrawal threshold in the contralateral hind paw of DKO mice was not significantly different from presurgical baselines (Figure 1A and B). The significant reduction of the withdrawal threshold of wild-type (WT) ipsilateral hind paw was dramatically ameliorated in the DKO mice (Figure 1B), suggesting that DKO mice exhibit a neuropathic pain-resistant phenotype. Since PRIP expression in WT mice was similar to that in the PSNL and sham-operated mice (Figure 1C), the onset of neuropathic pain was not induced by the change of PRIP expression.

**Figure 2:**
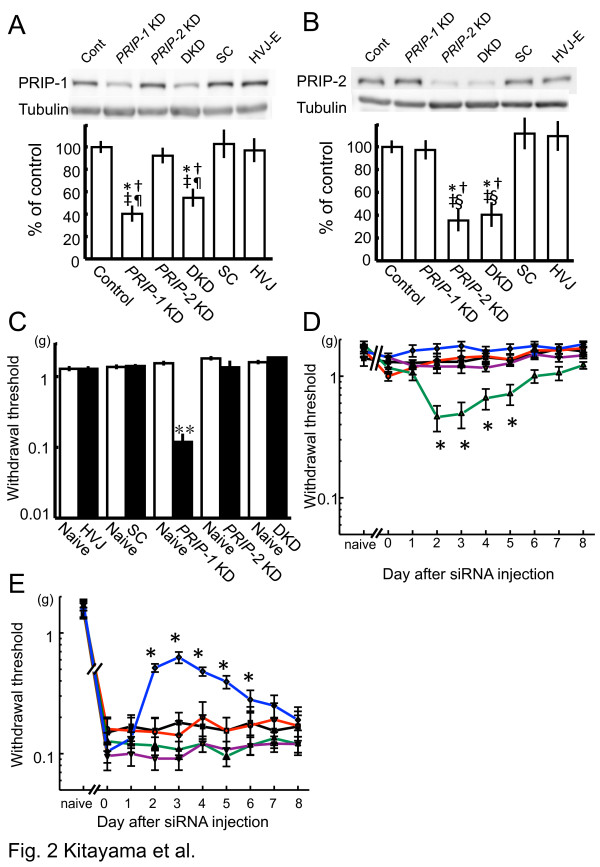
**Involvement of PRIPs in the pain-related behavior.** (**A**, **B**) Expression of PRIP-1 and PRIP-2 in the spinal cord of *PRIP-1* KD, *PRIP-2* KD and DKD mice 3 days after intrathecal siRNA injection. Immunoblot analyses were conducted using anti-PRIP-1 (**A**) and anti-PRIP-2 (**B**) antibodies. HVJ-envelope (HVJ-E) or scrambled siRNA (SC) was used as a negative control. For each, a representative image is shown in the upper panel. The level of immunoreactivity was normalized to β-tubulin and represented as % induction compared with the values of untreated mice (control) (means ± S.E.M., *n =* 4). *P < 0.05, **†***P* < 0.05, ‡*P* < 0.05, §*P* < 0.05 and ¶*P* < 0.05 compared with the corresponding values in untreated, SC, HVJ-E, *PRIP-1* KD, and *PRIP-2* KD mice, respectively (Tukey-Kramer test). (**C**) Influence on pain sensitivity by the suppression of the PRIP gene using intrathecal siRNA injection in mice. Paw withdrawal threshold was measured the day before (open column) and 3 days after (closed column) injection. Values represent withdrawal threshold (mean ± S.E.M., *n* = 5–10). **P < 0.01 compared with the corresponding values from before injection (Student’s *t*-test). (**D**, **E**) Influence on pain sensitivity by the suppression of the PRIP gene in PSNL-operated mice. Paw withdrawal threshold of both contralateral (**D**) and ipsilateral (**E**) sides were measured each day after the intrathecal siRNA injection (day 0). The values before the PSNL surgery represent as “naive.” Lines used in graphs are as follows: green, violet, blue, black, and red are for *PRIP-1* KD, *PRIP-2* KD, DKD, HVJ-envelope, and scrambled siRNA-injected mice, respectively. Values represent withdrawal threshold (mean ± S.E.M., *n* = 7). *P < 0.05 compared with the corresponding values of untreated mice (day 0) (Dunnet test).

To better understand the involvement of PRIP in nociceptive signaling, we produced the spinal cord-specific PRIP-1 knockdown (*PRIP-1* KD), PRIP-2 knockdown (*PRIP-2* KD), and PRIP-1 and PRIP-2 double knockdown (DKD) mice by using molecular specific siRNAs (Table 1) in the ddY mouse strain. We reported that a peak of gene suppression following intrathecal injection of a siRNA occurs at 2–3 days postinjection, and this recovers to original levels approximately 8 days after injection [14]. The significantly reduced expression of PRIP-1 in *PRIP-1* KD and DKD mice, or of PRIP-2 in *PRIP-2* KD and DKD mice, was observed 3 days after the siRNA injection (Figure 2A and B). We then examined mechanical allodynia by using the von Frey hair test in animals 3 days after siRNA injection. Allodynia was observed in the *PRIP-1* KD mice, but not in the other mice (Figure 2C), indicating that the *PRIP-1* KD mice mimicked the phenotypes of pain sensitivity observed in *PRIP-1* KO mice.

Next, we observed the influence of suppression of the *PRIP* gene on pain sensation by using a PSNL model. PSNL was performed on ddY mice 10 days before intrathecal siRNA injection, after which an allodynia score of contralateral and ipsilateral sides was analyzed during the 8 days after the injection of PRIP siRNA. In the contralateral paw, *PRIP-1* KD mice showed an allodynia in accordance with *PRIP-1* fluctuation 2–5 days after siRNA injection (initial score, 1.17 ± 0.09 at day 0; peak score, 0.46 ± 0.117 at day 2; and recovered score, 1.22 ± 0.08 at day 8) (Figure 2D). The PRIP-1 protein expression was analyzed by immunoblotting (data not shown). The allodynia observed in *PRIP-1* KD mice was not seen in *PRIP-2* KD, DKD, and other control mice (Figure 2D). However, the withdrawal threshold for the ipsilateral paw was dramatically increased in DKD mice (initial score, 0.104 ± 0.02 at day 0 and peak score, 0.628 ± 0.068 at day 3), but not other experimental mice, including *PRIP-1* KD and *PRIP-2* KD mice; the relief gradually reverted to painful levels within 7 days (Figure 2E). This suggested that suppression of both PRIP genes, but not either, induces resistance for pain sensation associated with allodynia.

**Figure 3:**
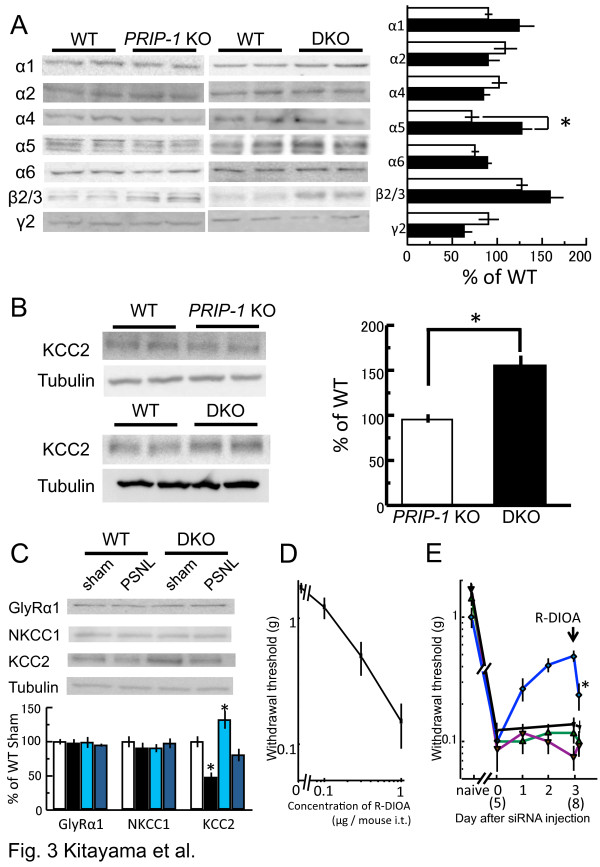
**Alteration of expression of GABA**_**A **_**receptor subunits and KCC2 in KO or KD mice.** (**A**) Expression of GABA_A_ receptor subunits in *PRIP-1* KO (open column) and DKO (close column) mice. Expression levels of *PRIP-1* KO and DKO are based on the corresponding WT (represented as 100%, *n* = 5). *P < 0.05, for values in DKO vs *PRIP-1* KO mice (Student’s *t*-test). (**B**) Expression of KCC2 in *PRIP-1* KO (open column) and DKO (close column) mice. Expression levels represent each corresponding WT value as 100%. *P < 0.05, for values in DKO vs *PRIP-1* KO mice (Student’s *t*-test). (**C**) Expression of GlyRα1 subunit, NKCC1 and KCC2 in WT and DKO 1 day after PSNL or sham operation (sham). The level of immunoreactivity was normalized to β-tubulin (mean ± S.E.M., *n =* 5–7). *P < 0.05, compared with values in WT sham-operated mice (Dunnet test). (**D**) R-DIOA administration induces pain relation behavior. Intrathecal administration of R-DIOA dose-dependently induced a decreased pain withdrawal threshold in WT mice. (**E**) Influence of KCC2 activity on pain sensitivity in PSNL mice. Each siRNA injection was performed 10 days after PSNL surgery. After 3 days of siRNA injection, R-DIOA (3 μg/mouse) was administrated intrathecally, and a paw withdrawal test was carried out 30 min after the injection. PSNL-operated WT mice were also treated with R-DIOA at 8 days after the surgery (representing as 8 in graph) and performed a paw withdrawal test. Naive represents prior to the surgery. Lines used in graph are as follows: black, green, violet, and blue are for WT, *PRIP-1* KD, *PRIP-2* KD, and DKD mice, respectively. The graph shows the withdrawal threshold value (mean ± S.E.M., *n* = 8). *P < 0.05, for values in 30 min after injection vs before injection (Student’s *t*-test).

Neuropathic pain in a model animal induces an altered expression of GABA_A_ receptors, including the downregulation of γ2 subunit-containing receptors [15,16]. PRIP is a modulator for GABA_A_ receptor intracellular trafficking [7,9,17]. The β 2/3 subunit is upregulated, and the γ2 subunit is downregulated in the spinal cord of *PRIP-1* KO or DKO mice [8,18]. Therefore, we examined the expression levels of GABA_A_ receptor subunits by immunoblotting using commercially available subunit-specific antibodies. The examined expressions were similar between the genotypes, with the exception of α5 expression, which was increased in DKO mice (Figure 3A). Knabel et al. reported that α2 and α3 contribute to diazepam-induced antihyperalgesia actions, but that α1 and α5 subunits do not [19], suggesting less involvement of spinal α5 subunit-containing GABA_A_ receptors in nociception [16]. Therefore, the different pain sensation between *PRIP-1* KO and DKO mice is probably not due to the alteration of GABA_A_ receptor expression in the spinal cord.

Inhibitory signaling is regulated by the intracellular chloride ion concentration, which is established in part via KCC2. Therefore, a high level of KCC2 expression drives chloride extrusion from neurons and maintains a low intracellular chloride ion concentration, i.e., GABAergic input may even acquire a net cell inhibitory response [20]. We next examined KCC2 expression by immunoblotting. The expression in *PRIP-1* KO spinal cord was similar to that in WT; however, the expression in DKO mice was significantly increased compared with WT and *PRIP-1* KO mice (Figure 3B). We then investigated the influence of PSNL surgery on expression of NKCC1 and KCC2, both of which are required for maintaining a fine balance between chloride ion influx and efflux, respectively (Figure 3C). KCC2 expression was higher in DKO sham-operated mice than in corresponding WT mice. PSNL surgery induced decreased KCC2 expressions in WT and DKO mice compared with sham-operated mice. Despite the decrease, KCC2 levels in DKO PSNL mice were similar to those in WT sham-operated mice. On the other hand, expression levels of NKCC1 were similar, and PSNL surgery did not affect the expressions (Figure 3C). We also examined expression of GlyR, contributing as a chloride ion channel dominantly expressing in the spinal cord, whose activation is known to ameliorates neuropathic pain [14]. Expressions of GlyRα1, a most prevalent subunit in central nervous system [21], were similar in the genotypes and were not changed by PSNL operation (Figure 3C). These data suggested that PRIP deficiency affects the expression of KCC2 at basal and after PSNL surgery. Since *PRIP-2* KO mice are not currently available, we were unable to define changes of GABA_A_ receptor, KCC2, NKCC2, and GlyRα1 expressions as a result of *PRIP-2* KO alone.

In immature neurons, a low expression of KCC2 results in a physiologically high concentration of intracellular chloride ions, which leads to the depolarization of cells [22]. Similarly, when PSNL was performed in mice, KCC2 expression was decreased in the spinal cord, resulting in a high concentration of intracellular chloride ion and reduced nociceptive threshold [23]. In addition, upregulation of KCC2 induced inhibitory postsynaptic potentials [24], suggesting that high expression level of KCC2 observed with DKO spinal cord enhances inhibitory synaptic transmission. We then tested if inactivation of KCC2 by R-(+)-[(dihydroindenyl)oxy] alkanoic acid (R-DIOA), an inhibitor of KCC2, affects pain sensitivity. The paw withdrawal threshold was decreased dose-dependently by intrathecal administration of R-DIOA (Figure 3D), indicating the importance of KCC2 activity. Therefore, similar KCC2 expression in the spinal cord of naive WT and DKO PSNL mice (Figure 3C) may cause the allodynia-resistant phenotype observed in the DKO PSNL model (Figure 1B).

To further confirm the involvement of KCC2 in neuropathic pain regulated by *PRIP*, we performed a hind paw withdrawal test by using WT, *PRIP-1* and *PRIP-2* KD, and DKD mice with R-DIOA. Withdrawal thresholds were not changed in PSNL-operated WT and a single gene KD mice, and R-DIOA administration did not affect the pain threshold (Figure 3E). However, relief from pain (as indicated by the increase of the threshold) in the DKD PSNL model was significantly inhibited by the administration of R-DIOA 3 days postinjection (Figure 3E).

## Conclusions

We demonstrated that the regular expression of KCC2 in DKO mice even after PSNL surgery induces the inhibition of nociceptive transmission and ameliorates PSNL-mediated neuropathic pain, even though the alteration of GABA_A_ receptor subunits in *PRIP-1* KO mice causes allodynia [8]. The current findings led us to hypothesize that regulation of KCC2 expression is a critical modulator of pain sensation.

## Abbreviations

DIOA: [(dihydroindenyl)oxy] alkanoic acid; DKD: *PRIP-1* and *2* gene double knockdown; DKO: *PRIP-1* and *2* gene double homologous knockout; GABA: γ-aminobutyric acid; GlyR: Glycine receptor; KCC2: K^+^-Cl^-^-cotransporter-2; NKCC1: Na^+^-K^+^-Cl^-^-cotransporter-1; PSNL: Partial sciatic nerve ligation; PRIP: Phospholipase C-related but catalytically inactive protein; PRIP-1 KO: PRIP-1 gene homologous knockout; WT: Wild type

## Competing interests

The authors declare that they have no competing interests.

## Authors’ contributions

TK carried out the paw withdrawal threshold test, immunoblotting, and data analyses. KM performed PSNL surgery and intrathecal siRNA injection. SR, NK, and KM participated in the data analyses. SU and MH provided the knockout mice and participated in the design of the study. TK conceived of the study, participated in its design and coordination of the experiments, and wrote the manuscript. All authors read and approved the final manuscript.
